# A Case of Ascending Colon Cancer Complicated by Cronkhite-Canada Syndrome Treated with Robot-Assisted Right Hemicolectomy

**DOI:** 10.70352/scrj.cr.25-0283

**Published:** 2025-10-02

**Authors:** Shigeyuki Hamanaka, Nobuki Ichikawa, Kengo Shibata, Tadashi Yoshida, Yosuke Ohno, Ken Imaizumi, Chihiro Ishizuka, Shunsuke Konishi, Akinobu Taketomi

**Affiliations:** Department of Gastroenterological Surgery I, Graduate School of Medicine, Hokkaido University, Sapporo, Hokkaido, Japan

**Keywords:** Cronkhite-Canada Syndrome, robot surgery, polyposis

## Abstract

**INTRODUCTION:**

Cronkhite-Canada syndrome (CCS) is a rare non-hereditary disorder in which multiple non-tumorous polyps occur throughout the digestive tract. Although surgical treatment is sometimes indicated due to the occurrence of gastric and colorectal cancer, in CCS, the polyposis makes it difficult to distinguish from cancer and requires careful follow-up. CCS is characterized by the presence of inflammatory findings, and steroids and immunosuppressants are used as key drugs. So, it is crucial to perform minimally invasive surgeries whenever possible, and in this regard, robotic-assisted surgery might prove to be one of the appropriate approaches.

**CASE PRESENTATION:**

The patient was a 72-year-old woman who had developed CCS 5 years previously and had been in remission with medication. During annual colonoscopy, multiple colorectal polyps were found in the ascending colon and resected endoscopically. One of the polyps was diagnosed as an adenocarcinoma with deep infiltration and vascular invasion. The patient was referred to our department for additional resection and a robotic-assisted right colectomy was performed. Pathological findings showed no residual tumor in the area after endoscopic mucosal resection (EMR), but laterally spreading tumor (LST) lesion showed adenocarcinoma in adenoma. The patient was discharged home on POD 7 without any postoperative complications.

**CONCLUSIONS:**

This report is the first case of robotic surgery for colorectal cancer complicated by CCS with a safe surgical outcome.

## Abbreviations

CCS
: Cronkhite-Canada syndrome
EMR
: endoscopic mucosal resection
LST
: laterally spreading tumor

## INTRODUCTION

CCS is a disease characterized by gastrointestinal polyposis and ectodermal abnormalities, which was first reported in 1995.^1)^ It is estimated to affect approximately one in one million people, and to date, approximately 500 cases have been reported worldwide, the majority of which are from Japan.^2)^ As the patient also had hypoproteinemia and inflammatory findings in the intestinal tract, minimally invasive treatment was required. Recently, minimally invasive surgery using robot-assisted surgery has been developed, and its application to colectomy is progressing in Japan. In the current case we performed robot-assisted right hemicolectomy for ascending colon cancer in a patient with CCS. This is the first report of robot-assisted colectomy for colorectal cancer with CCS.

## CASE PRESENTATION

**Fig. 1:**
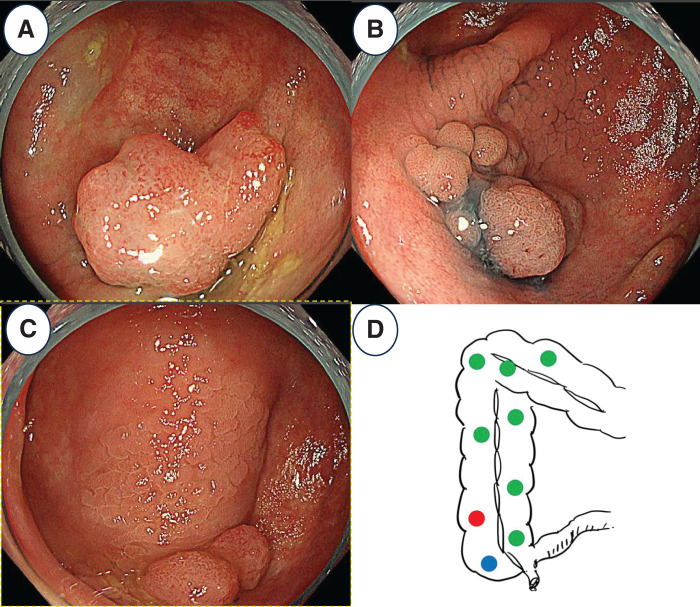
Colonoscopic findings. (**A**) Colonoscopy showed a tumor at ascending colon and the tumor was resected by EMR and had deep infiltration and vascular invasion. (**B**) A laterally spreading tumor near the cecum area. (**C**) Multiple polyps associated with CCS. (**D**) Schema of tumor and polyps distribution. The red dot is adenocarcinoma; The blue dot is the LST; green dots were multiple polyps. CCS, Cronkhite-Canada syndrome; EMR, endoscopic mucosal resection; LST, laterally spreading tumor

The patient is a 72-year-old woman. Five years ago, she was referred to our gastroenterology department with complaints of diarrhea, bloody stools, hair loss, and skin pigmentation. Endoscopy revealed multiple polypoid lesions, and serum albumin was less than 3.0 g/dL, indicating low levels. Treatment with prednisolone 30 mg/day was initiated for the diagnosis of CCS. After transitioning to a high-dose regimen of 60 mg/day of prednisolone, the patient achieved remission with azathioprine 75 mg/day following dose adjustment, and outpatient treatment continued. Even after achieving remission, annual colonoscopies revealed approximately 20 polyps each year, which were subsequently removed. During annual follow-up colonoscopy, multiple colorectal polyp lesions associated with CCS and signs of mucosal inflammation were noted from the cecum to the transverse colon. A 16-mm 0-Isp lesion was found in the ascending colon (**Fig. 1A**) and EMR was performed. The diagnosis was adenocarcinoma (A, type 0-Isp, 16 mm, tub1 >tub2, pT1b (SM; 1100 μm), BD2, Ly1c, V1a, pHM0 (1.5 mm), and pVM0 (800 μm), according to the Japanese Classification of Colorectal, Appendiceal, and Anal Carcinoma 3rd English Edition.^3)^ In addition, edema and inflammatory cell infiltration consisting mainly of mononuclear cells were observed around each polyp. The patient was referred to our department for additional resection because of the deep infiltration and vascular invasion in the tumor. At the time of her initial visit to our department, the patient was 146 cm tall, weighed 42.7 kg, and had a BMI of 20 kg/m^2^. At the initial visit, typical nail atrophy, pigmentation, and hair loss were observed as symptoms of CCS, but after treatment intervention, the symptoms showed a tendency to improve before surgery. Protein-losing enteropathy and diarrhea improved, but intestinal edema and inflammation associated with polyposis remained. Blood tests showed serum albumin at 4.2 g/dL, which was within the normal range. white blood cell (WBC) was 6300/µL and C-reactive protein (CRP) was 0.02 mg/dL, indicating no increase in inflammatory response. There were no metastases in the lymph nodes and distant organs on CT imaging, and the patient was diagnosed with Ascending colon cancer with cT1bcN0M0, cStage I. In addition, an LST was found in the cecum (**Fig. 1B**), and an adenoma was detected in a biopsy. In addition, there were multiple polyps in the area around the ascending colonic flexure (**Fig. 1C**, **1D**). The pathology revealed that the polyps were non-neoplastic. Blood tests were unremarkable.

**Fig. 2:**
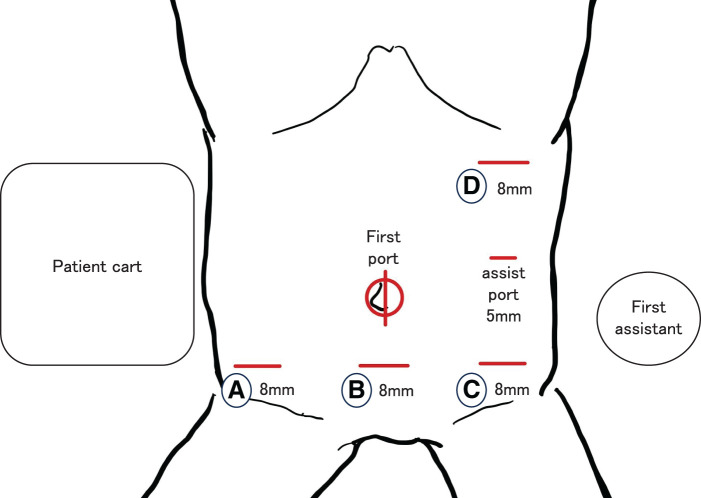
Setting port for right hemi-colectomy in our institute. (**A**) Fenestrated bipolar forceps. (**B**) 30-degree angle endoscope. (**C**) Monopolar curved scissors. (**D**) Tip-up fenestrated grasper. DaVinci Xi was positioned from the patient’s right side.

**Fig. 3:**
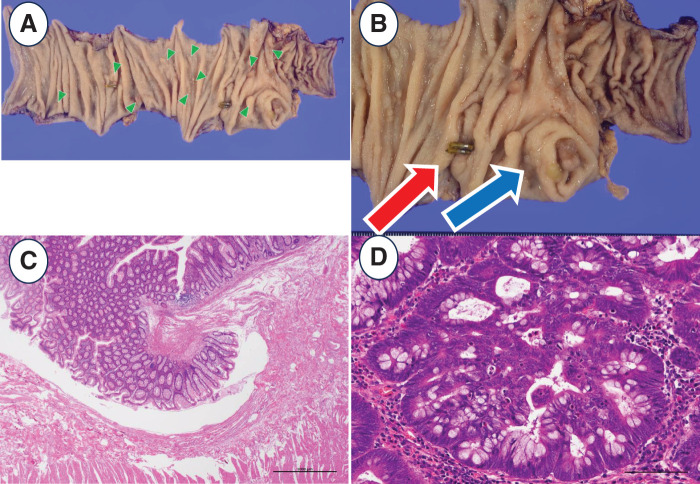
Pathological findings. (**A**) Clinical photograph of resected ascending colon. Green arrow heads are multiple polyps. (**B**) Expansion of (**A**). The red arrow is the clip of lesion indicated for additional resection. The blue arrow is LST lesion. (**C**, **D**) Histological specimens with H&E stain showed no residual tumor in the area after the EMR (**C**) and LST lesion showed adenocarcinoma in adenoma (**D**). EMR, endoscopic mucosal resection; LST, laterally spreading tumor

Surgery was performed using the DaVinch Xi. The operation was started with a 6-port configuration as shown in **Fig. 2**. The procedure, known as the squeezing approach, which has already been reported in laparoscopic surgery, was performed under robotic assistance.^4)^ In brief, after mobilizing the right half of the colon from the retroperitoneum, the omental bursa was opened and the accessory right colic vein was dissected from the cranial side. Finally, the ileocolic artery and vein were dissected at the root and robotic surgery was completed. A functional end-to-end anastomosis was performed to reconstruct the remaining intestinal tract outside the abdominal cavity. The operative time was 3 hours and 39 minutes, and there was no bleeding (0 mL). Pathological examination showed no residual tumor in the area after the EMR. The LST lesion showed adenocarcinoma in adenoma. There was no evidence of malignancy in the other polyps (**Fig. 3**). No steroids were administered during surgery. The patient resumed taking azathioprine orally on the 7th day. The patient started eating on POD 2 and was discharged home on POD 7 without any postoperative complications.

## DISCUSSION

The cause of CCS is unknown and it is considered to be a non-familial disease. It is more common in males (male-to-female ratio is 3:2) and the mean age of onset is 63.5 years (range 31–86 years). It is thought to be a chronic inflammatory disease related to autoimmune mechanisms^5)^ and presents with symptoms such as diarrhea, protein-losing enteropathy, hair loss, atrophy of the nail plate, and pigmentation.^6)^ Currently, immunosuppressive agents including corticosteroids are thought to be effective in approximately 80% of cases. Although the majority of CCS polyps are non-neoplastic, the prevalence of gastric and colorectal cancer has been reported to be from 10% to 20%.^6)^ Inflammatory polyposis in CCS is present throughout the gastrointestinal tract. Malignant polyps can occasionally be found coexisting with inflammatory polyps. However, it is difficult to detect malignant polyps and synchronous adenocarcinomas endoscopically at the initial presentation of CCS, because of multiple polyposis. Therefore, regular endoscopic follow-up is important and at least annual follow-up is recommended.^7)^ In cases such as the present one, aggressive resection can effectively remove cancer before its progression, proving to be a beneficial strategy. As mentioned above, CCS causes polyposis. In polyposis, there is a possibility of polyps becoming caught in the suture site, requiring caution during reconstruction. In this case, immediately prior to hospitalization for surgery, we requested marking of the most anal side of the transverse colon polyp using lower gastrointestinal endoscopy. During resection and reconstruction, we used external anastomosis and were able to determine the appropriate resection range by combining ink marking and palpation. CCS may be associated with the possibility of anastomotic leakage due to protein-losing enteropathy or intestinal edema. Egawa et al.^8)^ have investigated complications due to mucosal edema during gastric cancer surgery combined with CCS. In this case, although hypoalbuminemia had improved, inflammation around the polyp remained. Considering the possibility of suture failure as a postoperative complication, it was necessary to review the surgical procedure.

Robot-assisted colectomy has been reported to have similar outcomes as laparoscopic surgery, with a lower conversion rate to open surgery and shorter hospital stay, and it may be even less invasive.^9)^ In terms of short-term outcomes in colorectal cancer surgery, robot-assisted surgery has a lower incidence of complications, including anastomotic leakage, and has been shown to have clinical advantages, especially in right hemicolectomy.^10)^ In addition to the above, robot-assisted surgery has been shown to be non-inferior to laparoscopic surgery in terms of many complications. Robot-assisted surgery has been shown to be superior in terms of intestinal function recovery,^11)^ and has also been introduced for benign inflammatory bowel disease because of the advantage of postoperative outcomes. In surgical intervention for Crohn’s disease, an inflammatory disease that affects the entire gastrointestinal tract, patients who underwent robot-assisted surgery had a 14% lower rate of complications at 30 days postoperatively and a median hospital stay of 2 days shorter compared with open surgery.^12)^ Minimally invasive robot-assisted surgery is considered to be a good option even for patients with high-risk underlying diseases such as inflammatory bowel disease. CCS is characterized by the presence of inflammatory findings such as edema and redness even in the polyp-involved mucosa, and due to the nature of the disease, steroids and immunosuppressants are used as key drugs. Long-term use of steroids can increase vulnerability to stress and, in some cases, lead to postoperative adrenal insufficiency, infectious complications, or anastomotic leakage.^13)^ Kingham^14)^ reported that colonic anastomotic leakage in patients who used long-term steroids prior to surgery was significantly higher at 11.8% compared with 2.4% in non-users.

Therefore, it is crucial to perform minimally invasive surgeries whenever possible, and in this regard, robotic-assisted surgery might prove to be one of the appropriate approaches. There are currently few reports on surgical cases of colon cancer associated with CCS. However, considering the inflammation of the intestinal mucosa and the effects of drug therapy, it is important to select a surgical procedure that does not burden the patient. In this case, although the patient was in remission, inflammation of the intestinal mucosa remained. It is considered appropriate to have selected a minimally invasive surgical procedure. There are currently few reports on the usefulness of minimally invasive surgery for gastrointestinal cancer with concomitant CCS, so it will be necessary to collect more cases in the future. This is the first report of robotic-assisted surgery performed for the resection of colon cancer in a case of CCS.

## CONCLUSIONS

Robot-assisted surgery was performed safely for the case with colorectal cancer that had developed in CCS. In the case of CCS, early treatment of cancer could be achieved by annual screening and prompt resection of suspected neoplastic lesions or malignant tumors. In addition, minimally invasive surgery represented by robot-assisted surgery might be desirable for inflammatory diseases such as CCS.
